# Circular RNAs and Their Emerging Roles in Immune Regulation

**DOI:** 10.3389/fimmu.2018.02977

**Published:** 2018-12-18

**Authors:** Lan Yang, Jinrong Fu, Yufeng Zhou

**Affiliations:** ^1^Children's Hospital and Institute of Biomedical Sciences, Fudan University, Shanghai, China; ^2^Key Laboratory of Neonatal Diseases, Ministry of Health, Shanghai, China

**Keywords:** circRNA, biogenesis, function, research approaches, immune regulation

## Abstract

Circular ribonucleic acid (RNA) molecules (circRNAs) are covalently closed loop RNA molecules with no 5′ end caps or 3′ poly (A) tails, which are generated by back-splicing. Originally, circRNAs were considered to be byproducts of aberrant splicing. However, in recent years, development of high-throughput sequencing has led to gradual recognition of functional circRNAs, and increasing numbers of studies have elucidated their roles in cancer, neurologic diseases, and cardiovascular disorders. Nevertheless, studies of the functions of circRNAs in the immune system are relatively scarce. In this review, we detail relevant research on the biogenesis and classification of circRNAs, describe their functional mechanisms and approaches to their investigation, and summarize recent studies of circRNA function in the immune system.

## Introduction

Circular RNA (circRNA) is a covalently closed loop molecular form of RNA, and was discovered more than two decades ago (1, 2). Initially, circRNA was considered to be an aberrant byproduct of splicing (3–6). Recently, numerous circRNAs have been identified as a consequence of rapid developments in bioinformatics and high-throughput sequencing. Jeck et al. detected >25,000 circRNAs in fibroblasts using a genome-wide RNase R enrichment strategy (7). Memczak et al. identified 1950 circRNAs in humans, 1903 in mice, and 724 in *Caenorhabditis elegans* using RNA-sequencing data combined with analyses of the human leukocyte database (8). circRNAs are also expressed in fungi, plants, and protists (9–12).

Current research on circRNAs focuses on their role in cancer, neurologic diseases, and cardiovascular disorders. In this review, we cover relevant research on the biogenesis and classification of circRNAs and their functional mechanisms and methodological approaches to their study, along with summarizing recent investigations of the roles of circRNAs in the immune system.

## Biogenesis of circRNAs

**Figure 1:**
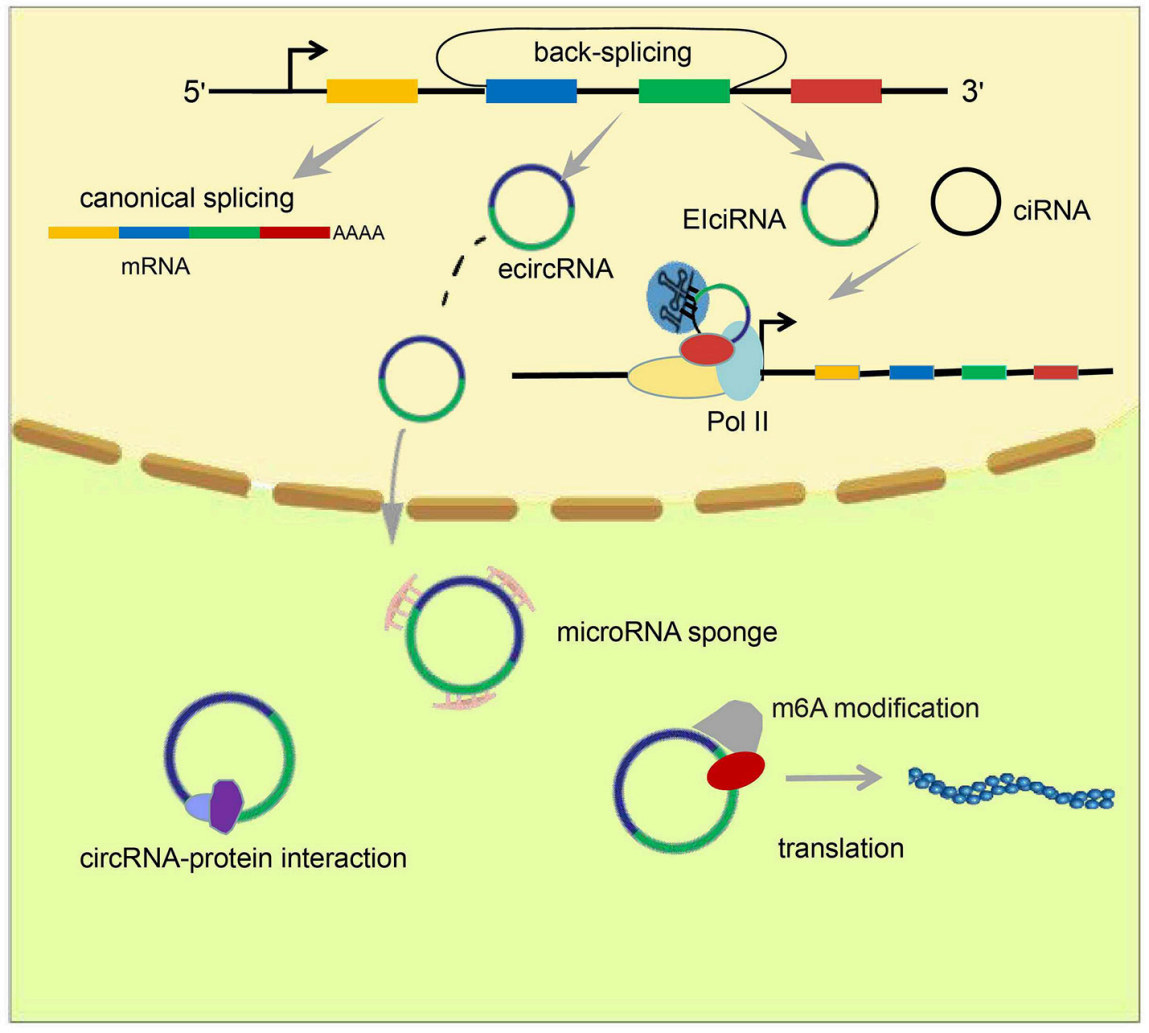
Formation and classification of circRNAs and models of circRNA function. circRNAs are formed by back-splicing. There are three major types of circRNA: ecircRNA, ciRNA, and EIciRNA. EcircRNAs function mainly in the cytoplasm through the “microRNA sponge” mechanism, whereas EIciRNA and ciRNA accumulate in the nucleus and facilitate transcription of their parent genes *via cis*-regulatory effects. In addition, some circRNAs have been reported to act by interacting with or encoding proteins.

Rather than canonical splicing, circRNAs are generated through back-splicing (13, 14) (Figure 1). Back-splicing can be accompanied by transcription (15) or may occur after transcription has been completed (16). Three main mechanisms have been reported to produce circRNAs: exon skipping, intron pairing-driven circularization, and RNA binding protein (RBP)-driven circularization.

### Exon Skipping

Skipping of exons leads to the formation of a lariat structure containing the skipped exon. If introns are spliced before the lariat is unraveled by debranching enzymes, a stable exon-containing circRNA will be produced (7), along with a linear transcript excluding the skipped exon(s). This mechanism was an early understanding of how circRNAs are formed because the linear transcripts that are produced alongside circRNAs were described some years ago (17, 18).

### Intron Pairing-Driven Circularization

Intron pairing-driven circularization is based on reverse complementary matches (RCMs) within flanking introns, and was established independently of the exon-skipping mechanism of circularization. RCMs can induce base-pairing between flanking introns, promoting a hairpin formation, bringing the 5′ and 3′ termini of an exon into spatial proximity, and inducing “head-to-tail” splicing. Intriguingly, the protein adenosine deaminases acting on RNA (ADAR) also participate in this process, together with RCMs. ADAR can unzip double-stranded RNA molecules by converting adenosine residues within them into creatinine molecules, thereby reducing the formation of circRNAs (19). Furthermore, Jeck et al. determined that, unlike sequence regions involved in generation of non-circular transcripts, the 200 bp upstream or downstream of a back-splice site contain canonical complementary ALU repeats, suggesting that intronic pairing may drive the circularization of exonic circRNAs (ecircRNAs). Jeck et al. also found that the length of introns flanking ecircRNAs are greater than those flanking linear RNA exons (7).

### RBP-Driven Circularization

In addition to the involvement of introns and exons, some RNA-binding proteins also contribute to the formation of circRNAs. For example, in *Drosophila*, the splicing factor muscleblind (MBL) drives the circularization of the second exon of *MBL* to produce circMBL, by binding specifically to MBL binding sites within introns flanking circMBL sequences (15). More importantly, the interaction between MBL and circMBL contributes to regulation of the levels of MBL protein. When MBL is present in excess, it reduces the production of *MBL* mRNA by promoting circMBL formation. A protein called “quaking” (QKI) was identified by Conn et al. and shown to promote the production of circRNAs during human epithelial–mesenchymal transition by binding to a motif within circRNA flanking introns (20). RBM20 is also an RNA-binding protein that regulates numerous cardiac-specific gene-editing processes. Mutation of RMB20 is involved in dilated cardiomyopathy through its effects on the generation of circRNAs from *Titin* (21). The RNA-binding protein FUS can mediate circRNA formation by mediating RNA back-splicing in neurons (22). Moreover, HNRNPL, an RNA splicing factor, participates in regulation of circRNA formation in prostate cancer (23). In conclusion, the formation of circRNAs is dependent upon the regulation of cis elements and trans-factors (24).

## circRNA Classification

Studies have identified three main types of circRNA: ecircRNA, circular intronic RNA (ciRNA), and exon–intron circRNA (EIciRNA) (Figure 1). Published data suggest that ecircRNAs function mainly through the microRNA “sponge” mechanism, proposed first by Memczak et al. who found there were 63 microRNA-7 binding sites on CDR1as, and hence designated CDR1as a “microRNA sponge.” EcircRNA can enhance levels of microRNA target genes through adsorption of microRNA molecules. Unlike ecircRNAs, intron-containing circRNAs (ciRNAs or EIciRNAs), in general, reside in the nucleus and regulate gene transcription (25–27). Chen et al. found that the function of ci-ankrd52, which is derived from the second intron of *ANKRD52*, may depend on a consensus motif containing a 7-nucleotide (nt) GU-rich element near its 5′ splice site and an 11-nt C-rich element close to the branchpoint to avoid being debranched. Ci-ankrd52 accumulates mainly in the nucleus and promotes transcription of *ANKRD52* through a *cis*-regulatory effect of RNAPol II (25).

## circRNA Characteristics

circRNAs are not degraded readily by RNase R, and are more stable than their cognate linear mRNAs, with half-lives exceeding 48 h (7). circRNAs also exhibit a degree of conservation between species. Jeck et al. identified ecircRNAs from 14.4% of actively transcribed genes in human fibroblasts. Furthermore, they identified 69 ecircRNAs from murine testes that mapped to precisely orthologous chromosome locations compared with human circRNAs (7).

Numerous circRNAs exhibit specificity to tissue, developmental period, or cell type. Memczak et al. found that different cells express specific circRNAs through identification of circRNAs in four human cell types: cluster of differentiation (CD)19^+^ leukocytes, CD34^+^ leukocytes, neutrophils, and HEK293 cells. Analogously, they noted that several nematode circRNAs were expressed in oocytes but absent in 1- or 2-cell embryos (8).

Although most circRNAs do not have the potential for translation (28), several recent studies have confirmed that circRNAs can be translated into proteins. Legnini et al. screened differentially expressed circRNAs in Duchenne muscular dystrophy by RNA sequencing. They determined that circ-ZNF609 can affect muscle formation through regulation of myoblast proliferation. Surprisingly, they also found that circ-ZNF609 can encode a protein (29), but whether this protein has a role in myoblast proliferation is not known. Yang et al. demonstrated that modification of *N*^6^-methyladenosine (m6A), which is induced by the methyltransferase METTL3/14 and suppressed by the demethylase FTO, promotes protein translation through recruiting an initiation factor, eIF4G2, and a m6A reader, YTHDF3. Furthermore, they found that m6A-containing circRNAs, with the potential to be translated into proteins, are common in the human transcriptome (30). Yang et al. also demonstrated that circ-FBXW7 can be translated into the protein FBXW7-185aa, thereby co-regulating the stability of c-Myc, along with its parent gene-encoded protein, FBXW7, to inhibit the progress of malignant glioma (31). Pamudurti et al. described the discovery of numerous circRNA-translated proteins or peptides based on ribosome “footprinting” experiments in *Drosophila* brains. They demonstrated that ribosomes can bind at the stop codon of circMbl and that a protein encoded by circMbl can be identified by protein spectroscopy (32).

Several reports have detailed many chemical modifications present on DNA and RNA. Surprisingly, recent breakthrough investigations of chemical modification of circRNAs have demonstrated that a m6A modification occurs in circRNA. This was discovered first by Yang et al. who further demonstrated that this modification promotes protein translation through recruitment of the initiation factor eIF4G2, and the m6A reader YTHDF3 (30) (Figure 1). Zhou et al. further confirmed the existence of the m6A modification in circRNAs, and also proposed some characteristics of m6A-modified circRNAs (33).

## Mechanisms of circRNA Function

### MicroRNA Sponge

MicroRNAs produced by Dicer processing enzymes from single-stranded RNA precursors with a hairpin structure of ~70–90 nt are single-stranded small RNAs of ~21–23 nt (34, 35). Through initiation of RNA-induced silencing complex (RISC), mRNAs are degraded or their translation is hindered by base pairing with target gene transcripts (36, 37). Memczak et al. was the first to propose the microRNA sponge model. They found that there were 63 microRNA-7 binding sites within the circular transcript CDR1as (ciRS-7). Hansen et al. determined that a testes-specific circRNA called Sry can also serve as a sponge for microRNA-138 (38). Recent studies have shown that various circRNAs can function to adsorb microRNAs, thereby releasing target mRNAs (Figure 1). This process is associated with several diseases. In 2017, Han et al. demonstrated that circMTO1 upregulates p21 by competitively binding to microRNA-9, thereby inhibiting the proliferation of hepatocellular carcinoma (39). Zhong et al. clarified that the circRNA MYLK acts as a competing endogenous RNA (ceRNA) to bind directly to microRNA-29a, thereby promoting the development of bladder cancer by activating VEGFA/VEGFR2 signaling pathway and the Ras-Erk pathway (40). The circRNA circEPSTI1 was discovered to be an endogenous competitive RNA and sponge for microRNAs. Through binding to microRNA-4753 and microRNA-6809, circEPSTI1 upregulates expression of B cell CLL/lymphoma 11A (BCL11A), promotes the proliferation of triple-negative breast cancer cells, and inhibits their apoptosis (41).

### Interactions Between circRNAs and Proteins

Rather than acting as microRNA sponges in the cytoplasm, some circRNAs act by interacting with corresponding proteins (Figure 1). In 2016, circ-foxo3 was found to impede cell-cycle progression by forming a ternary complex with p21 and Cdk2 proteins (42). In addition, another circRNA, circ-Amotl1, which is highly expressed in cancerous cell lines, can increase nuclear retention of the oncogenic protein c-myc to promote its stability, and increase its affinity for binding to several promoters, thereby upregulating c-myc targets such as HIF1α, Cdc25a, ELK-1, and JUN (43). This observation reveals a novel function of circRNAs in tumorigenesis. Conceivably, there are circRNAs other than circ-Amotl1 that act by similar mechanisms. In 2017, Abdelmohsen et al. was the first to report competition between a circRNA and its cognate mRNA for the RNA-binding protein HuR, which has been studied extensively and can regulate protein expression through interaction with a wide range of RNAs. They proposed that high levels of circPABPN1, a circRNA derived from *PABPN1*, repress HuR binding to PABPN1 mRNA through binding to HuR itself, causing a reduction in PABPN1 mRNA translation (44). This mechanism, based on the interaction between a circRNA and its cognate mRNA, provided new insights and spurred further investigation of the roles of circRNAs in the nucleus.

### Regulation of Transcription in *cis*

Multiple non-coding RNAs, including HOTAIR and MALAT1, are known to regulate gene transcription in *trans*; that is, they influence the transcription of genes other than their parent genes. However, there have been studies that have shown circRNAs, mainly intron-containing circRNAs (ciRNAs or EIciRNAs), can have *cis*-regulatory effects on gene expression. In 2013, Chen et al. reported that ci-ankrd52, which is generated from the second intron of ANKRD52, accumulates primarily in the nucleus and promotes transcription of ANKRD52 *via* the *cis*-regulatory effects of RNAPol II (25). In 2014, Li et al. proposed a model for the *cis*-regulatory effects of EIciRNAs based on their findings from investigation of two EIciRNAs: circEIF3J and circPAIP2. They proposed that EIciRNAs may interact with proteins such as U1 snRNP *via* RNA–RNA interplay between U1 snRNA and EIciRNA. Then, EIciRNA–U1 snRNP complexes could interact with Pol II at the promoter regions of parental genes, thereby enhancing their transcription (Figure 1). This phenomenon produces a positive feedback loop because, once transcription has been initiated, EIciRNA generation will increase, further promoting gene transcription (26).

### circRNAs as Biomarkers

In addition to being detected inside cells, circRNA has also been reported to be present in extracellular fluids. Li et al. enriched exosomes in serum samples from patients suffering from colon cancer to examine circRNA expression in exosomes. Compared with healthy controls, hundreds of circRNAs were expressed differentially in serum exosomes from patients with colon cancer (45). In 2016, Guarnerio et al. was the first to report that chromosomal translocations can produce fusion circRNAs in acute promyelocytic leukemia. Furthermore, using an MLL/AF9-AML model, they found that f-circM9 could contribute to progression of acute myeloid leukemia (46). Based on those findings, Tan et al. confirmed that the common fusion gene in non-small-cell lung cancer (NSCLC), *EML4-ALK*, can produce a fused circRNA called F-circEA, and demonstrated that F-circEA promotes the proliferation and migration of cells. F-circEA was also detected in the serum of *EML4-ALK-*positive patients, indicating that this circRNA is highly likely to be useful as a diagnostic marker for *EML4-ALK*-positive NSCLC (47). With further research it is likely that disease-specific circRNAs will be developed as disease biomarkers.

## Approaches to Studies of circRNA

### Identification Tools of circRNA

Identification of circRNA after sequencing is the first step in circRNA research. Various identification tools have been developed. Whether the identification of circRNAs is accurate and comprehensive is dependent upon the rigor and reliability of the algorithm. The Find_circ algorithm uses bowtie2 to map the original reads to the reference genome, discards all mapped sequences, takes 20 nt of each unmapped read as an anchor, and then determines the location of the anchor in the genome again to identify whether the splicing of circRNA is present (8). The CIRCexplorer algorithm uses the TopHat algorithm to map the RNA-sequencing reads to the human hg19 reference genome, and then maps the unmapped reads with the TopHat-Fusion algorithm. Such reads, unmapped with TopHat but mapped with TopHat-Fusion on the same chromosome in a back-spliced order, are extracted as candidate back-spliced junctionreads (48). The CIRI algorithm proposes paired chiastic clipping (PCC) signals to identify circRNAs. The PCC signal is detected by collecting and comparing the alignment information of all the segments of a read. PCC signals do not rely on existing annotation information, so back-splicing can be identified from zero to predict various types of circRNA, including intronic and intergenic circRNAs (49). In 2015, Hansen et al. found that short circRNAs (especially circRNAs < 200 nt in length) are degraded readily by RNase R, and that a circRNA predicted by a single algorithm specifically has a higher false-positive rate. Conclusively, they suggested that identification of circRNAs can be done using a combination of different tools or RNA libraries with linear RNA being removed (50). We have summarized the four commonly used circRNA algorithms in Table 1.

**Table 1:** Identification algorithms for circRNA.

**Tool**	**Mapper**	***De novo*?**	**Annotation information?**
Find_circ	Bowtie2	Yes	No
CIRCexplorer	Bowtie1 and 2	No	Yes
CIRI	Bwa	Yes	No
Mapsplice (51)	Bowtie1	No	Yes

### Enrichment and Verification of circRNAs

RNase R is a member of the *Escherichia coli* RNR superfamily. It can cleave RNA in the 3′-5′ direction and digest almost all linear RNAs, but it cannot digest circRNAs readily (52). High-throughput sequencing for genome-wide identification of circRNAs requires enrichment of circRNA through treatment of samples with RNase R to remove linear RNAs, followed by enhancement of the concentration of circRNAs to facilitate their identification. Two approaches are used for the identification of circRNAs: reverse transcription-polymerase chain reaction (RT-PCR) and northern blotting. In RT-PCR, after digestion by RNase R, cDNA samples are amplified with divergent primers and convergent primers. Subsequent agarose-gel electrophoresis reveals that amplification with divergent primers generates a band in the RNase R(+) group, whereas the convergent primers produce no band. Divergent and convergent primers generate bands in the RNase R(–) group, indicating that circRNAs are present and resistant to digestion by RNase R (31, 53). Northern blotting is conducted using probes specific for circRNA and mRNA. The results show that linear mRNA cannot be detected in the RNase R(+) group, whereas its corresponding circRNA is visible, indicating that mRNAs are digested, whereas circRNAs are not (54). Notably, some circRNAs will also decrease in abundance after long-term digestion by RNase R, probably because of susceptibility to RNase R.

### Knockdown and Knockout of circRNAs

Technology based on RNA interference is used widely to knockdown circRNA expression. To eliminate non-specific knockdown effects on cognate linear RNA, a specific small interfering RNA (siRNA) or short-hairpin RNA (shRNA) targeting circRNA must be directed to the back-splicing site. This strategy limits the design of siRNA sequences, and siRNA or shRNA targeting back-splicing sites will be partially complementary to the cognate linear RNA, raising the possibility of unwanted effects on expression of linear RNA. To solve this problem, Li et al. proposed that the control sequence should be partially replaced (~10 nt) by a siRNA sequence targeting the back-splicing site (24). Similarly, knockout of circRNAs in animals risks simultaneously influencing expression of the cognate linear RNA and completely knocking out a gene is highly likely to affect expression of neighboring genes (55, 56). Based on the mechanism of circRNA production, Zhang et al. achieved knockout of circGCN1LI in human PA1 cells by removing an intron complementary sequence using CRISPR/Cas9 (16).

### circRNA Overexpression

Plasmids used commonly to overexpress circRNAs are universal loop-forming framework vectors and gene-specific flanking sequence vectors. For example, Liang et al. cloned the exon2/3 and flanking sequences of *ZKSCAN1* to construct a vector, and then overexpressed circ-ZKSCAN1. Then, the flanking sequence was modified, and exons2/3 of ZKSCAN1 was replaced with a polyclonal restriction site to construct an empty vector: pcDNA3.1(+) CircRNA Mini Vector (57). Overexpression using a gene-specific flanking vector sequence is consistent with generation of natural circRNAs *in vivo*. Based on the characteristics of circRNA flanking sequences, length-appropriate flanking repeats (Alu elements) and circRNA sequences are cloned to generate a eukaryotic expression vector construct. In 2015, Lu et al. found that tRNA introns can form circular RNAs (tricRNAs) (58) and, in 2016, Schmidt et al. created a new circRNA expression vector based on tRNA splicing and transformation. First, they designed restriction enzyme sites in the two bulge–helix–bulge (BHB) regions of the *tRNA* intron and inserted the circRNA sequence for expression (59). In brief, the vector produced a circRNA by joining BHB motifs *via* an RtcB enzyme, which was not dependent upon a reverse-complementary sequence.

In general, different circRNAs with different loop-forming characteristics exhibit variation in looping efficiency. Moreover, circRNA production by overexpression vectors is accompanied by the production of linear isoforms, which is an additional challenge for strategies employing circRNA overexpression.

## circRNAs in Immunity

### circRNAs in Anti-virus Immunity

**Figure 2:**
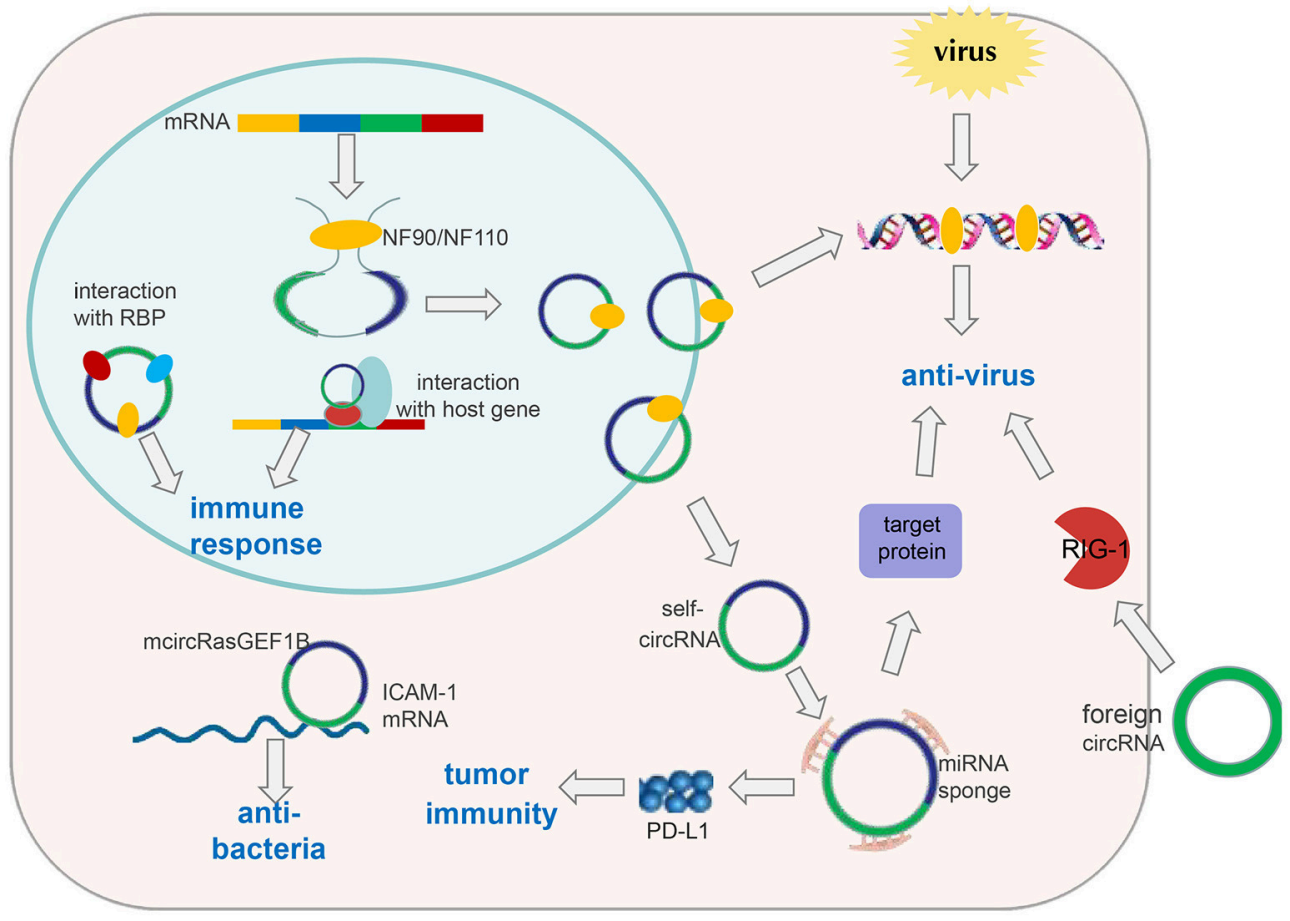
CircRNAs in immune responses. Exogenous circRNAs can activate the RIG-1 cellular immune response pathway to suppress viral replication. The immune factor NF90/NF110 can promote circRNA formation by stabilizing the intron complementary sequence. Under viral infection, NF90/NF110 is exported from the nucleus to the cytoplasm to inhibit virus replication. Meanwhile, circRNA expression in the nucleus is decreased. Thus, endogenous circRNAs can be used as “molecular indicators” of antiviral proteins to prompt antiviral immune responses. circRNAs can also function as “microRNA sponges” to increase expression of target proteins involved in antiviral responses or tumor immunity, such as PD-L1. mcircRasGEF1B can help to protect cells from bacterial infection by enhancing the stability of mature ICAM-1 mRNA. In addition, circRNAs can be involved in immune responses by interacting with proteins or their host genes.

Few reports have elucidated the role of circRNA in immune responses. Nevertheless, Chen et al. attempted to construct circRNAs to transfect cells based on *in vitro* transcription followed by auto-splicing circulation. Surprisingly, they found that *in vitro* circRNAs induced activation of cellular immune response pathways and inhibited RNA virus infection, which is mediated by retinoic acid-inducible gene-I (RIG-I); however, endogenous circRNAs did not induce this pathway because of their binding to specific RNA-binding proteins (60) (Figure 2). These findings suggest that exogenous introduction of circRNAs could be used to activate antiviral immune responses for therapeutic purposes. Nevertheless, some interesting questions must be solved: How do RBPs recognize self and non-self circRNAs, and then induce activation of non-self circRNAs on the RIG-1 pathway?

In addition, Li et al. found that circRNAs are involved in viral infection *via* the immune response factor NF90/NF110 (61). First, they applied a genome-wide siRNA screening strategy targeting all unique human genes with a Dox-inducible circmCherry expression vector to profile proteins involved in circRNA biogenesis. Consequently, they determined that NF90/NF110, encoded by the interleukin enhancer binding factor 3 (*ILF3*) gene, promoted circRNA formation by stabilizing the intron complementary sequence. Upon viral infection, NF90/NF110 was exported from the nucleus to the cytoplasm to inhibit virus replication. Meanwhile, circRNA expression in the nucleus was decreased (Figure 2). In this way, circRNAs may be used as “molecular indicators” of NF90/NF110 to prompt antiviral immune responses. However, the detailed mechanisms by which antiviral proteins promote circRNA biogenesis and key elements within circRNAs to interact with antiviral proteins have yet to be explored.

The two studies mentioned above provide evidence to support circRNA involvement in antiviral immunity through interactions with specific antiviral proteins (62). However, one of the studies elucidated the function of exogenous circRNAs in antiviral immunity, and the other study suggested endogenous circRNAs may be used as molecular indicators of antiviral proteins to prompt antiviral immune responses. In addition to interacting with antiviral proteins, circRNAs may have other roles during antiviral immune responses. Through sequencing of the whole transcriptome, Shi et al. found that expression of 536 circRNAs was dysregulated significantly in herpes simplex virus 1 (HSV-1) -infected cells in contrast to uninfected human fibroblasts. Similarly, they screened differentially expressed genes and microRNAs in HSV-1-infected cells. Furthermore, they undertook analyses of Gene Ontology (GO) and Kyoto Encyclopedia of Genes and Genomes (KEGG) databases. Their results suggested that these differentially expressed genes were very enriched in the pathways of immune responses, such as the NOD-like receptor signaling pathway and JAK-STAT signaling pathway (63). An integrated analysis of the circRNA–microRNA–gene axis revealed circRNAs can regulate the genes associated with host immune responses, which was mediated by microRNAs. These data suggest that circRNAs can regulate host antiviral immune responses through interactions with the corresponding microRNAs (Figure 2). However, these results based on large-scale bioinformation analyses should be validated experimentally.

Interestingly, several studies have demonstrated that some viruses can encode their own microRNAs, which facilitates the entry, replication, and virulence of viruses by targeting host transcripts, including some antiviral signaling molecules (64–67). For example, microRNA-BHRF1-3 encoded by the Epstein–Barr virus diminishes the levels of CXC-chemokine ligand 11, a chemoattractant in immune responses (68). In recent years, ceRNAs, including long non-coding RNA and circRNA (which can combine with microRNAs completely with a protein-coding target) have been studied widely. Ghosal et al. established a database named “HumanViCe” in which circRNAs that can sponge virus microRNAs can be predicted, and these circRNAs are enriched in pathways associated with the entry and replication of viruses and host immune responses (69). In summary, HumanViCe can aid exploration of the roles of circRNAs in viral infection but also circRNAs may act as potential antiviral targets.

### circRNAs Acting Against Bacterial Infections

Ng et al. identified a lipopolysaccharide (LPS)-inducible circRNA generated from linear RasGEF1B, named mcircRasGEF1B, the expression of which is dependent upon the LPS-Toll-like receptor-4-nuclear factor-kappa B (LPS-TLR4-NF-κB) pathway. mcircRasGEF1B is cell type-specific, exhibits evolutionary conservation between mice and humans, and localizes preferentially to the cytoplasm (70). Also, its knockdown using the corresponding antisense oligodeoxynucleotide (ASOs) reduces intercellular adhesion molecule (ICAM-1) expression in the LPS/TLR4 signaling pathway by affecting the stability of the mature ICAM-1 mRNA, but not mRNA splicing (70) (Figure 2). In the immune system, ICAM-1 recruits leukocytes to sites of tissue inflammation, as well as enhancing adhesion between antigen-presenting cells and T cells (71, 72). In addition, ICAM-1 has been reported to inhibit M2 polarization of macrophages in tumors (73). Therefore, we speculate that mcircRasGEF1B may contribute to suppression of polarization of M2 macrophages during immune responses. These discoveries broaden our understanding and suggest that circRNAs may be important for the “fine tuning” of immune responses, and may help to protect cells from microbial infection.

### circRNA as a ceRNA in Tumor Immunity

The relationship between microRNAs and immunity has been well-studied, leading to the hypothesis that circRNA may contribute to immune regulation through interactions with microRNAs. Zhang et al. reported that hsa_circ_0020397 can upregulate expression of PD-L1 (the target gene of microRNA-138) by binding to microRNA-138 in colorectal cancer cells. The consequent increase in PD-L1 levels contributes to tumor escape from immune responses (74, 75) (Figure 2). This information provides new insights for “checkpoint therapy” in cancer patients. Zheng et al. demonstrated that circHIPK3 rescues the downregulation of microRNA-124 on expression of interleukin (IL)-6R (76), implying that circHIPK3 may function in tumor immunity response. There are several bioinformatics databases that can be used to predict circRNAs that could bind to microRNAs, including circbase, starBasev2.0, and circinteractome (77).

### circRNAs Regulate Immunity *via* Proteins

In addition to microRNAs, circRNAs can interact directly with proteins that function in immune responses. Similarly, we can predict such circRNAs using bioinformatics databases, then verify the predictions using *in vitro* and *in vivo* experiments. Using the circinteractome database, we found that the host gene of hsa_circ_0032139 is *HIF1A*, which plays an important part in inflammation *via* NF-κB and mitogen-activated protein kinase (MAPK) pathways (78). Under hypoxia, degradation of HIF1A protein is prevented, leading to its accumulation, and association with HIF1B to exert transcription regulation on target genes, including pro-inflammatory cytokines, most glycolytic enzymes, and glucose transporters, among others (79–83). Presumably, hsa_circ_0032139 can regulate inflammation through its association with *HIF1A*. Another circRNA, hsa_circ_0038481, has been predicted to associate with *TLR4*, a classical pattern-recognition receptor. This implies that hsa_circ_0038481 may be involved in LPS-stimulated signaling pathways, such as the NF-κB and MAPK.

### circRNAs in Immune-Related Diseases

Rheumatoid arthritis (RA) is a chronic, inflammatory synovitis-based systemic disease of unknown etiology (84). Zheng et al. screened the top-ten upregulated, and downregulated circRNAs in RA patients based on analyses of peripheral-blood mononuclear cell chips, and selected the top-five corresponding microRNAs for each circRNA (85). We can speculate that these differentially expressed circRNAs may function in RA by acting as sponges of the corresponding microRNAs, which have been reported to be associated with RA. More importantly, the study by Zheng et al. provides clues that circRNAs may have the same effects as their host genes in RA. For example, hsa_circ_0038644, one of the dysregulated circRNAs in RA, is spliced from *PRKCB*, which is associated with LPS-induced activation of the NF-κB signaling pathway (86). Therefore, we hypothesized that hsa_circ_0038644 can aggravate inflammation in RA patients.

Type-2 diabetes mellitus (T2DM) is characterized by hyperglycemia, insulin resistance, and chronic inflammation (87). Fang et al. found circANKRD36 to be upregulated markedly in the peripheral blood cells of T2DM patients. Furthermore, an association was noted between circANKRD36 expression and inflammatory factors (88). Therefore, circANKRD36 may serve as a potential biomarker and be involved in inflammation in T2DM.

Similarly, Li et al. measured circRNA expression in the T cells of patients with systemic lupus erythematosus (SLE). They found hsa_circ_0045272 to be downregulated significantly. Furthermore, they demonstrated that hsa_circ_0045272 regulated apoptosis and IL-2 production negatively (89). However, the mechanisms underlying the involvement of hsa_circ_0045272 in SLE pathogenesis merits further exploration.

Numerous studies have shown that a high proportion of tumor-infiltrating lymphocytes (TILs) in the tumor microenvironment can improve clinical outcomes. Weng et al. showed that high expression of hsa_circ_0064428 is associated with a low proportion of TILs, poor survival, large tumor volume, and tumor metastasis in patients with hepatocellular carcinoma (90). These observations suggest that hsa_circ_0064428 can act as a potential immune-associated prognosis biomarker for hepatocellular carcinoma.

Overall, the roles of circRNAs in immune diseases have been studied based on large-scale microarray and RNA sequencing analyses, by which differentially expressed circRNAs were screened to further verify their functions experimentally. However, the mechanisms by which circRNAs regulate disease development merit further exploration.

## Perspectives

Although circRNAs have become a hot topic in RNA research in recent years, several important areas merit investigation. For example, Dong et al. showed that circRNA can be reverse-transcribed *in vivo* and fused into the genome to generate a pseudogene (91). Further research is needed to elucidate the molecular mechanisms underlying circRNA *trans*-transcriptional translocation and the effects of pseudogenes derived from circRNAs. In addition, unlike linear mRNA, construction of overexpression vectors for circRNA is challenging because it requires splicing of the two termini of amplified fragments to generate loop structures. Moreover, data in the mouse circRNA database is incomplete, which poses specific obstacles for *in vivo* experiments. Finally, circRNAs in the cytoplasm, which act as microRNA sponges, have been studied widely, but circRNAs that can function through this mechanism are in the minority. Most circRNAs rarely contain so many miRNA binding sites and have low expression (24, 28), so future research should explore other mechanisms of circRNAs.

Few studies have focused on circRNAs in the immune response. Reminiscent of studies in cancer, circRNA research in immunity also requires measuring circRNA expression, and then elucidating the function of differentially expressed circRNAs and the mechanisms underlying it. Notably, circRNA localization could aid exploration of the mechanisms by which circRNAs regulate immune responses. circRNAs in the cytoplasm are likely to act as microRNA sponges, whereas circRNAs in the nucleus are likely to interact with proteins, or elicit effects by promoting/suppressing the role of their host genes (Figure 2). Excitingly, circRNAs are promising markers or drug targets for some immune diseases, which could facilitate rapid diagnosis and treatment.

## Author Contributions

LY the first author, contributed to collection of references and manuscript preparation. JF and YZ contributed to manuscript modifications.

### Conflict of Interest Statement

The authors declare that the research was conducted in the absence of any commercial or financial relationships that could be construed as a potential conflict of interest.
